# An artificial intelligence deep learning model for identification of small bowel obstruction on plain abdominal radiographs

**DOI:** 10.1259/bjr.20201407

**Published:** 2021-04-27

**Authors:** DH Kim, H Wit, M Thurston, M Long, GF Maskell, MJ Strugnell, D Shetty, IM Smith, NP Hollings

**Affiliations:** 1 The Department of Clinical Imaging, The Royal Cornwall Hospitals NHS Trust, Truro, UK; 2 The Medical Imaging Department, University Hospitals Plymouth NHS Trust, Plymouth, UK; 3 The Department of General Surgery, The Royal Cornwall Hospitals NHS Trust, Truro, UK

## Abstract

**Objectives:**

Small bowel obstruction is a common surgical emergency which can lead to bowel necrosis, perforation and death. Plain abdominal X-rays are frequently used as a first-line test but the availability of immediate expert radiological review is variable. The aim was to investigate the feasibility of using a deep learning model for automated identification of small bowel obstruction.

**Methods:**

A total of 990 plain abdominal radiographs were collected, 445 with normal findings and 445 demonstrating small bowel obstruction. The images were labelled using the radiology reports, subsequent CT scans, surgical operation notes and enhanced radiological review. The data were used to develop a predictive model comprising an ensemble of five convolutional neural networks trained using transfer learning.

**Results:**

The performance of the model was excellent with an area under the receiver operator curve (AUC) of 0.961, corresponding to sensitivity and specificity of 91 and 93% respectively.

**Conclusion:**

Deep learning can be used to identify small bowel obstruction on plain radiographs with a high degree of accuracy. A system such as this could be used to alert clinicians to the presence of urgent findings with the potential for expedited clinical review and improved patient outcomes.

**Advances in knowledge:**

This paper describes a novel labelling method using composite clinical follow-up and demonstrates that ensemble models can be used effectively in medical imaging tasks. It also provides evidence that deep learning methods can be used to identify small bowel obstruction with high accuracy.

## Introduction

Small bowel obstruction is a common surgical emergency accounting for approximately 15% of acute surgical admissions and over 12,000 major emergency operations a year in England and Wales alone.^1–3^ The most common causes include adhesions, hernias and malignancies.^4^ Delay in the diagnosis and surgical management of small bowel obstruction increases the risk of bowel necrosis, perforation and death and therefore timely diagnosis is critical to improve patient outcome.^5–7^

Although CT is a more sensitive test, the plain abdominal radiograph is frequently used as a first-line screening tool in the hospital setting^1,8,9^ as it is cheap, quick and easily available. The hallmark of acute small bowel obstruction on plain radiograph is the presence of disproportionately dilated small bowel compared with large bowel. Several additional radiographic features of small bowel obstruction have also been described, including the stretch sign, absence of rectal gas, string-of-beads sign and a gasless abdomen.^4,10^

Radiologist staffing levels vary worldwide but in countries such as the United Kingdom, there is currently a severe shortage of consultant radiologists and this has resulted in reporting backlogs in recent years.^11^ The COVID-19 pandemic has caused a shift in this trend recently, but it is likely that reporting demand will outpace capacity in the future particularly as imaging departments attempt to clear the backlog of imaging requests delayed as a result of the pandemic. The availability of immediate expert radiologist review of plain X-rays is therefore variable and some departments will be forced to prioritise immediate reporting of other modalities such as emergency CT. In such instances, the first person to review an abdominal radiograph may be a junior doctor who is not always adequately trained in X-ray interpretation. We know that the accuracy of plain radiographs for detecting small bowel obstruction varies significantly with the experience of the interpreter.^12^ Due to the reasons mentioned above, it is not uncommon for the formal radiology report to be delayed for several hours in some institutions. Artificial intelligence (AI) has the potential to address some of these issues since automated image review could occur immediately at the time of image acquisition, flagging high-risk cases for expedited radiology or surgical review.

In recent years, AI in the form of deep learning has been applied to numerous medical imaging tasks and in some cases the solutions are considered to be as accurate as an expert human interpreter.^13,14^ Deep learning utilises neural networks which are computational models comprising multiple layers of interconnected nodes which process packets of data before passing the data to the next layer of the network. The final layer of the network is an output layer that can be used to make predictions. At and between each node, the data are processed according to parameters that are not explicitly programmed but learnt by the network over several iterations according to training data.^15^

In automated medical imaging interpretation tasks, convolutional neural networks are the current state-of-the art. Numerical data are derived from image pixel values by sliding, or ‘convolving’, a window across all areas of the image. Image features are identified by the network and the importance of each feature is defined by the learning process. In early layers, simple image features such as lines and textures predominate but these simple features are effectively combined in later layers allowing the network to ‘perceive’ greater levels of complexity and abstraction.

Training convolutional neural networks typically requires very large data sets which are often difficult to obtain in the clinical setting. However, it is possible to adopt a neural network that has been pre-trained with large quantities of data and adapt it for use in a different task where data may be less abundant. This process is called transfer learning. This method utilises the ability of the pre-trained network to identify common image features that may be applicable to both tasks, such as simple shapes or textures.^16^

This study aimed to investigate the extent to which transfer learning with convolutional neural networks could be used to identify small bowel obstruction on plain abdominal radiographs. If successful, this technique could be used at the point of X-ray acquisition to alert the radiologist or surgeon to cases with high probability of small bowel obstruction. This would expedite clinical review and potentially result in faster diagnosis and treatment.

## Methods and materials

This study was approved by the Health Research Authority, UK. Ethics approval was not required as the study was limited to the use of pre-existing, anonymised data.

### Data acquisition and labelling

Plain abdominal radiographs were obtained from the Royal Cornwall Hospitals NHS Trust, United Kingdom. The X-rays were obtained with the patient in the supine position. Images were exported in de-identified JPEG format from the picture archiving and communication system. The inclusion period was January 2010 to January 2019. Studies were excluded if the patient was under 18 years of age, if the patient had received oral contrast, if the indication was not for the investigation of suspected bowel pathology or if the radiograph demonstrated large bowel obstruction.

Images were labelled at the time of export as either “normal” or “obstruction”. Labelling was achieved primarily via inspection of the consultant radiologist report. If the report was inconclusive, then composite clinical follow-up was used for labelling. This comprised assessment of any subsequent CT imaging or surgical operation notes in the following 72 h, and if necessary, inspection of the hospital discharge summary.

If studies remained indeterminate after composite clinical follow-up, the image underwent enhanced review. A subset of 25 indeterminate images was independently reviewed by three consultant (FRCR) UK Radiologists with a subspecialty interest in gastrointestinal (GI) imaging. The unanimous opinion was used as a subjective threshold to label images that were initially indeterminate. Cases that remained indeterminate after enhanced radiological review were excluded, since no meaningful label could be assigned.

The final data set comprised a total of 990 labelled images, 445 normal and 445 demonstrating small bowel obstruction.

### Training and testing data

**Figure 1.**
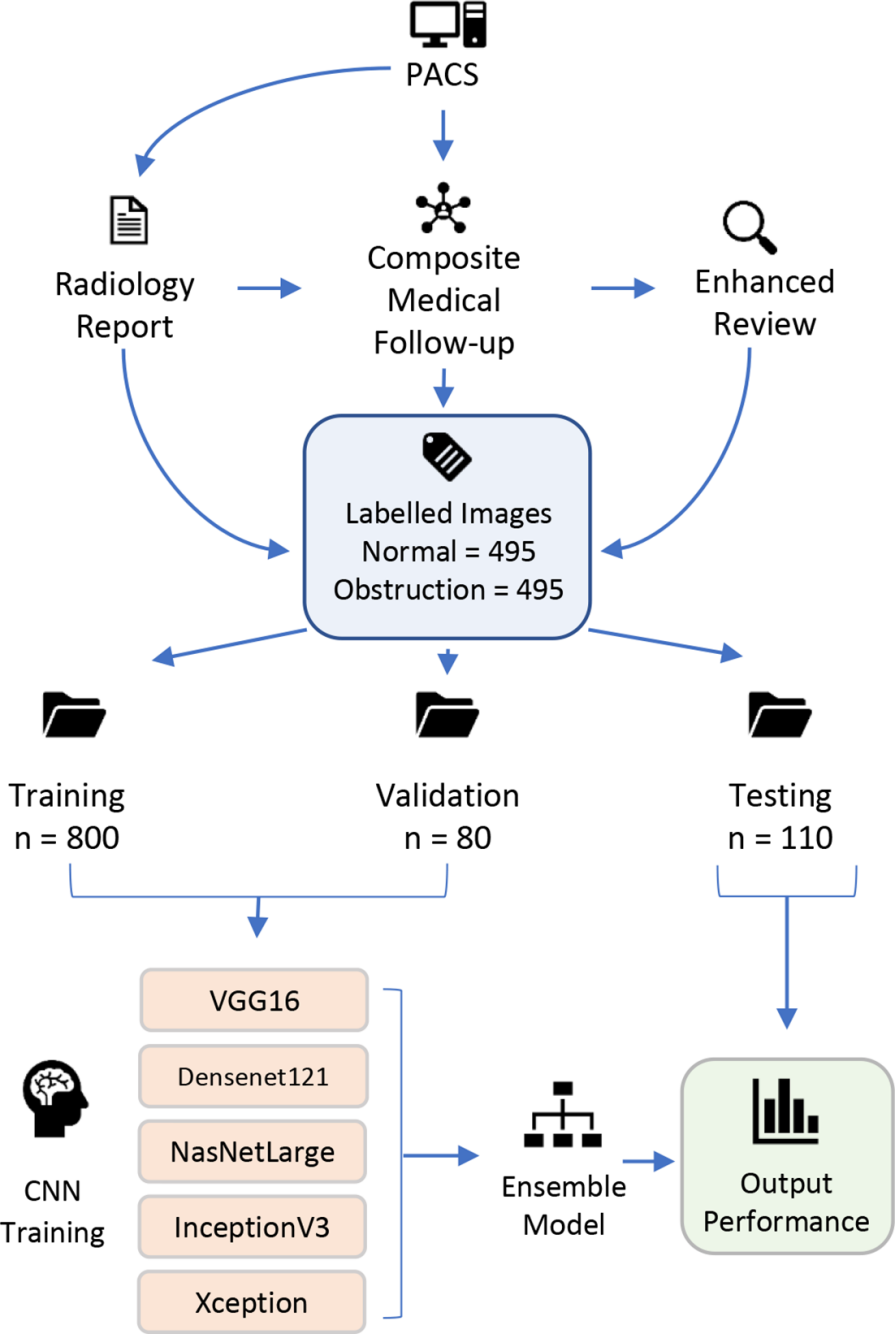
Flow diagram illustrating image labelling and network training methods. CNN, convolution neural network; PACS, picture archiving and communication system

Imaging data were split into training, validation and test sets using a ratio of 80:8:11 respectively. Images were allocated randomly to each group. The training data set comprised the majority of the data and was used to teach the neural networks. The validation data set was used to guide the network after each learning cycle to ensure it was updating itself correctly. The test data set was used only once, after the network had been trained, to assess final performance. This data split resulted in 800 training images, 80 validation images and 110 testing images (Figure 1). This ratio was designed to maximise training data while maintaining adequate statistical power in final performance testing. A test set sample size of 110 enabled a statistical power of 0.8 for detecting an AUC of 0.65 with a Type 1 error of 0.025.^17^ This meant that the study was sufficiently powered to evaluate a single diagnostic test with an AUC anywhere between 0.65 (poor diagnostic test) and 1.0 (perfect diagnostic test). The test data were not used at any point in training or validation.

### Network architecture

CNNs were developed using the Keras library^18^ in Python (v. 3.7.3) programming language with TensorFlow backend.^19^ All neural networks consisted of a pre-trained base layer beneath a fully connected top layer. The output was a score of between 0 and 1 with scores closer to 1 indicating a higher computational probability of small bowel obstruction. Dropout at a rate of 0.5 was used between the fully connected and output layers. This process randomly removes nodes from the network during training to help reduce the reliance of a network on any single node. This creates redundancy in the network making the network more generalisable to unseen data. A dropout of 0.5 was chosen, as it is considered close to optimal for a wide range of networks and tasks.^20^

Five architectures were employed, namely: VGG16, Densenet121, NasNetLarge, InceptionV3, and Xception.^21–25^ These are publicly available network architectures which were initialised with weights pre-trained on the ImageNet data set^26^ without the top layers and with all lower layer weights frozen. This means the foundations of the networks were developed to detect everyday objects such as vehicles, flowers or animals. The final layers were completely new and learnt their parameters based on the radiographs used in training (*i.e.* transfer learning).

Training images were resized according to the default image size of the base network, varying in size from 224 × 224 to 331 × 331, with pixel values rescaled to between 0 and 1. This was performed using the Keras pre-processing ‘flow_from_directory’ method based on nearest neighbour interpolation. Networks were trained on a desktop machine comprising a Nvidia GeForce RTX 2070s (8 GB) Graphics Processing Unit, a 6-core Intel i5-9400F CPU (2.90 GHz) and 16 GB RAM.

### Network training and testing

Hyperparameters are the settings used in network training. These were optimised using an iterative process using the validation loss as a guide to model performance. Validation loss is the error within the network and demonstrates how well the network is performing. The hyperparameters included: the type of optimiser (the algorithm used to minimise the error), the learning rate (the magnitude of changes made to network parameters at each iteration) and the learning rate reduction (used to refine the learning rate as training progresses to avoid over shooting the optimum solution). Networks were trained until no further improvement was seen in the validation loss. The network with the lowest validation loss was saved (*i.e*. the network with the best performance). Further details of the specific hyperparameters used can be found in Supplementary Material 1.

Supplementary Material 1.

The performance of each network was then assessed using the previously unused test data. This consisted of presenting each test case to the neural network model and asking the model to make a prediction as to whether the case demonstrated small bowel obstruction or not. The output format was a computational confidence value for each case of between 0 and 1 for the presence of small bowel obstruction. An ensemble model was developed using the mean confidence value derived from the output of the five networks.

The AUC for each network and for the ensemble model was calculated in Python using the scikit-learn library.^27^ AUC confidence intervals and statistical comparisons were calculated using the “easyROC” v. 1.3.1 software, utilising a non-parametric approach.^17^ The Youden J index can be calculated for each operating point and is equal to sensitivity + specificity −1. The operating point with the highest Youden J index was selected for reporting sensitivity and specificity values. This is the point at which sensitivity and specificity are maximised.

A Densnet121 network with weights initialised from a network trained on the ChexNet data set^28^ was also developed. CheXNet is a large data set of chest radiographs. The hypothesis was that these weights may be more transferable to a task involving abdominal radiographs than weights trained on ImageNet. However, the validation loss was comparatively poor and therefore this model was not included in the ensemble model. Training was also performed with data augmentation in which the training data were amplified by small changes in rotation (0–10 degrees), height/width dimension (0–20%), zoom (0–20%) and shear (0–5%). Lastly, a variety of training image sizes were compared using the VGG16 network.

## Results

**Figure 2.**
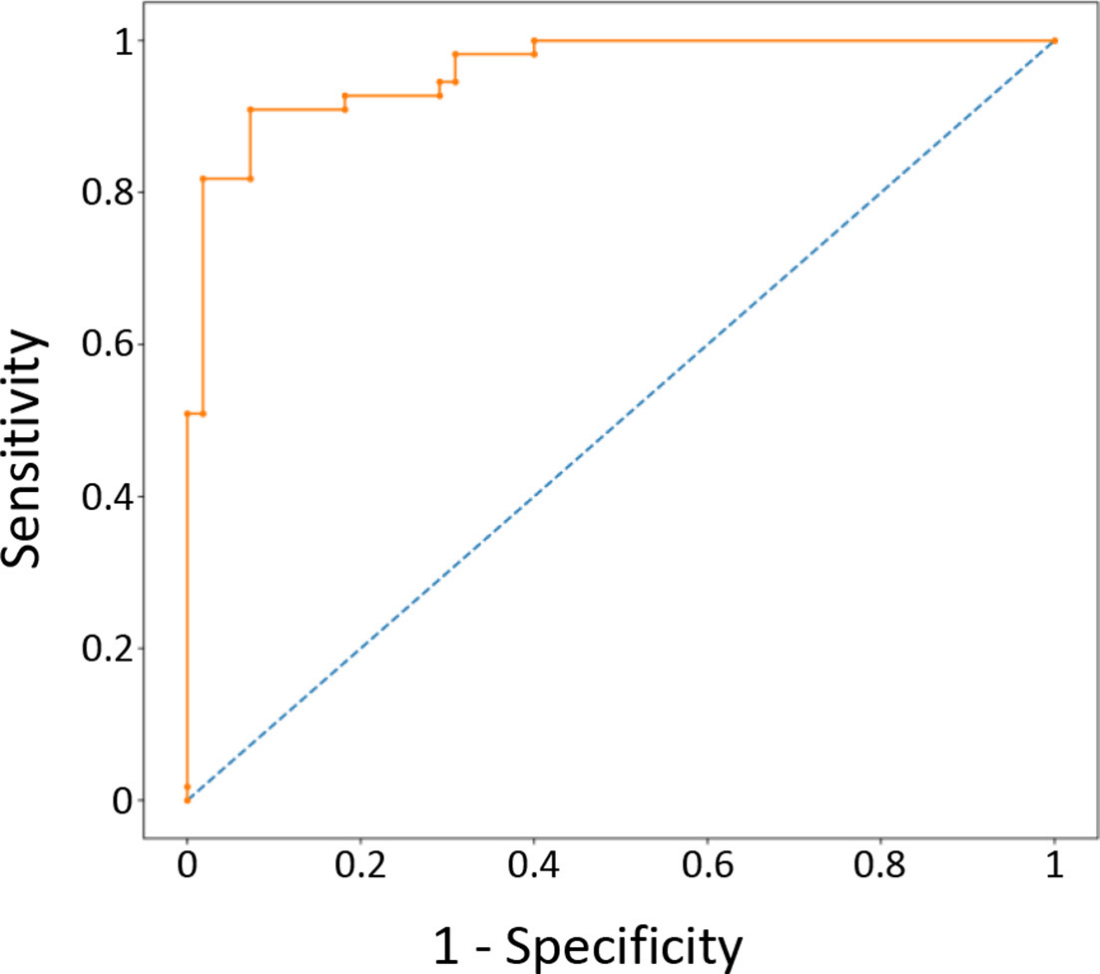
ROC curve for the ensemble model. ROC, receiver operator characteristic.

The ensemble model demonstrated excellent performance on the test set with an AUC of 0.961 (Figure 2). The operating point with the highest Youden J index yielded a sensitivity of 91% and specificity of 93% (Table 1). Once loaded into memory, the ensemble model provided a prediction in approximately 146 ms per image. The ensemble model produced the highest accuracy and AUC metrics but there was no statistically significant difference between the ensemble and the individual networks based on a limited testing sample. The AUC values for each individual network are shown in Table 2. Training plots are depicted in Supplementary Material 2.

Supplementary Material 2.

**Table 1.** Performance of the ensemble model

	True normal	True SBO	Total
Test normal	51	5	56
Test SBO	4	50	54
Total	55	55	

SBO, small bowel obstruction.

**Table 2.** Test metrics for the five neural networks and ensemble model

Base CNN model	Validation loss	Test loss	Test accuracy	AUC^*a*^
VGG16	0.269	0.468	0.86	0.918 (0.864–0.971)
Densenet121	0.21	0.368	0.85	0.929 (0.884–0.974)
NasNetLarge	0.179	0.416	0.88	0.934 (0.887–0.981)
InceptionV3	0.262	0.278	0.88	0.953 (0.918–0.988)
Xception	0.231	0.372	0.85	0.925 (0.872–0.977)
Ensemble			0.92	0.961 (0.929–0.992)

AUC, area under the receiver operator characteristic curve; CNN, convolutional neural network.

a AUC. The 95% confidence intervals are shown in brackets.

**Figure 3.**
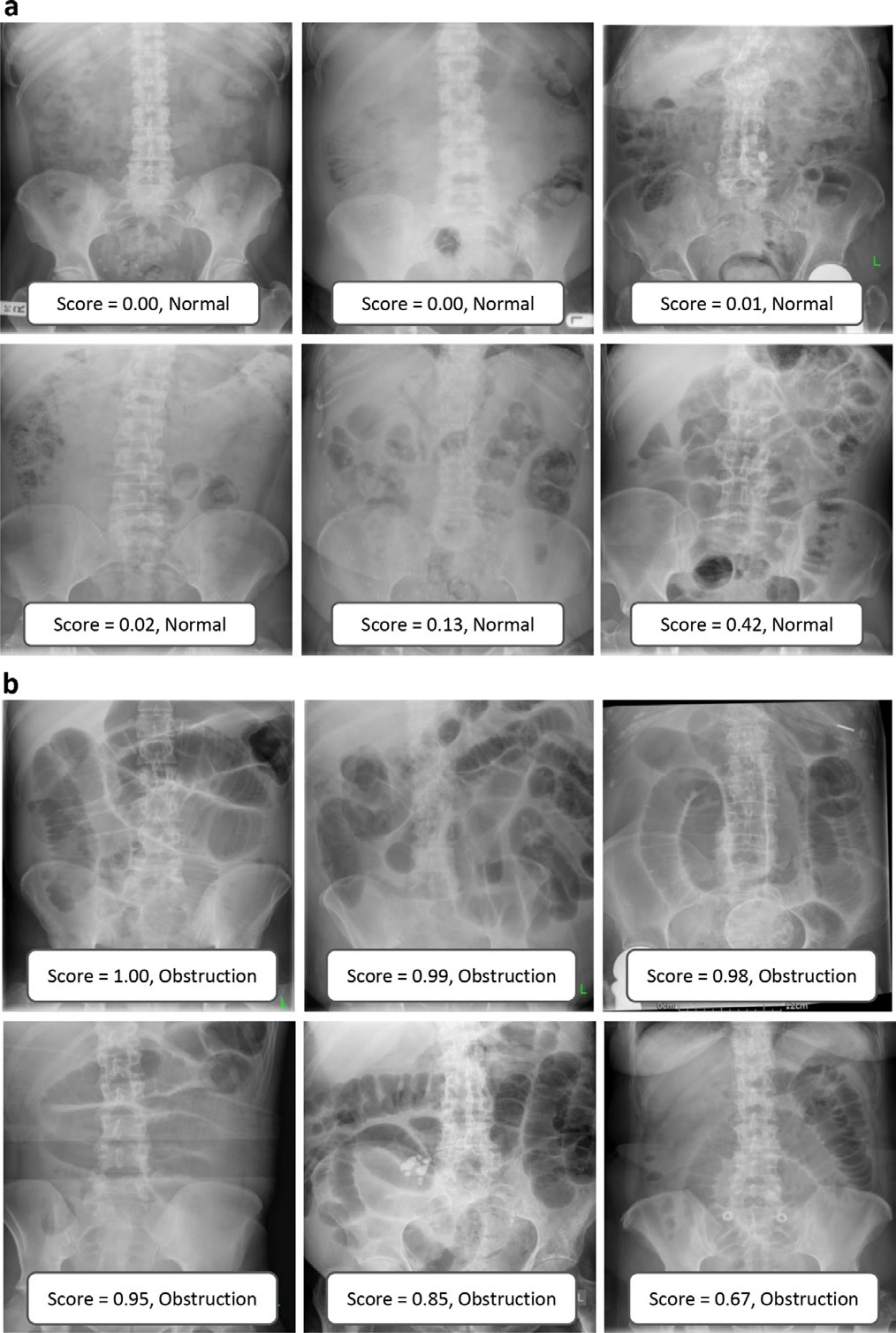
Examples of correctly categorised images demonstrating normal appearances (a) and obstruction (b). The ensemble model scores and category predictions are shown in boxes at the base of each image.

**Figure 4.**
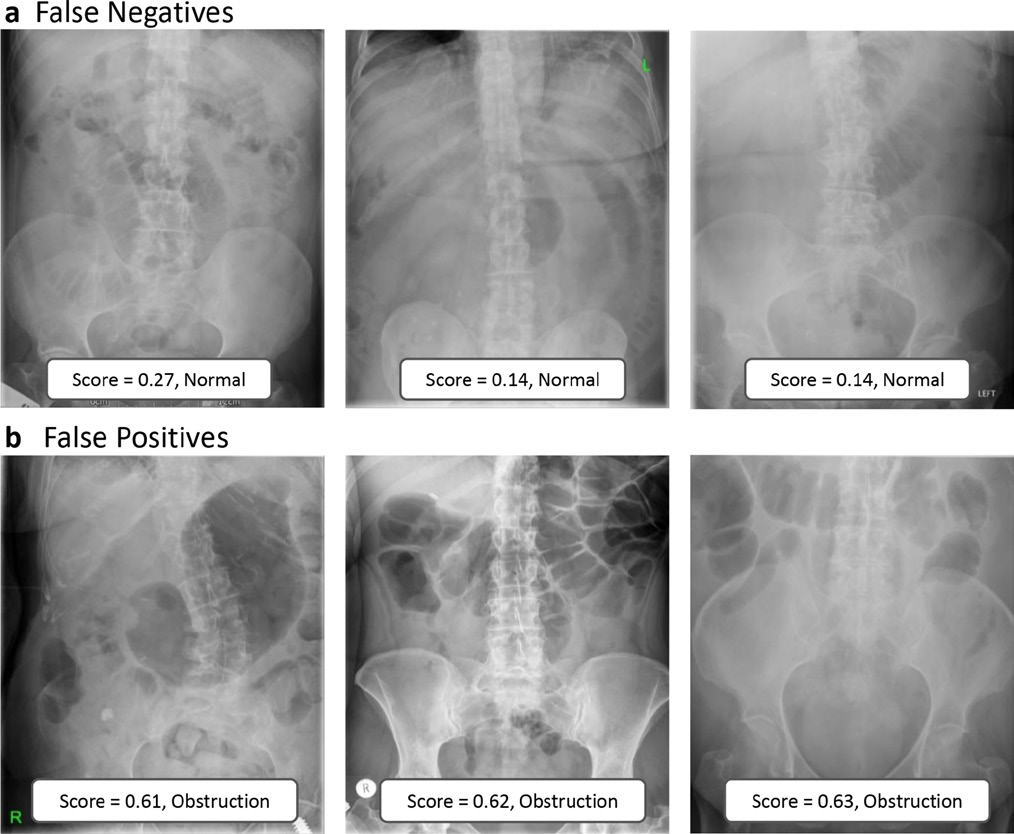
Examples of incorrectly diagnosed images showing false negatives (a) and false positives (b). Ensemble model output scores and class predictions are shown in boxes at the base of each image.

Examples of correctly and incorrectly categorised images are shown in Figures 3 and 4. Qualitative inspection of the misclassified images suggests that the neural networks struggled with gas-filled large bowel and a gas-filled stomach.

Data augmentation did not result in improved network accuracy. Inspection of the training log demonstrated that training with the augmented data resulted in greater overfitting of the model as suggested by the increased discrepancy between training and validation loss. Training the network with different sized images also provided no performance benefit. Lastly, training a Densenet121 network with weights initialised from the CheXNet data set produced a comparatively poor result with a validation loss of 0.43.

## Discussion

The deep learning method described here produced excellent results and demonstrated that transfer learning can theoretically be applied to the identification of small bowel obstruction on plain abdominal radiographs. Furthermore, the excellent accuracy metrics detailed here suggest that this technique may be accurate enough to be practically useful in the clinical setting. For example, a tool such as this could be integrated into the clinical pathway by almost instantaneously providing a test score for an abdominal radiograph at the point of acquisition. High test scores could trigger an alert within the Radiology Information System, highlighting cases to be reported immediately by a radiologist, effectively eliminating the common problem of report lead-time delay. An electronic alert could also be sent to a member of the surgical team, prompting expedited review of the patient’s clinical status in correlation with the imaging. This has the potential for faster diagnosis and improved patient outcomes although this would need to be validated in the context of a prospective trial.

To the best of the authors knowledge, there are currently no commercially available solutions able to diagnose small bowel obstruction on X-ray. Furthermore, the literature on this topic is extremely limited.^29–31^ The two papers by Cheng et al^29,30^ are iterations of the same project. Both the latest version by Cheng et al^29^ and the study described here produced excellent and comparable results. Additionally, the current study has introduced a new ensemble method which demonstrated the highest accuracy and AUC metrics compared with the individual networks. Although the improvement in AUC was not statistically significant at the 95% level, the results were encouraging. Whilst the study was powered appropriately for a single diagnostic test, the authors suspect that a larger testing sample may be required to identify a statistically significant difference between the models, particularly given that the magnitude of any improvement necessarily diminishes as test accuracy approaches 100% . Further investigation in this area is necessary since this ensemble method could be widely applied to other medical imaging categorisation tasks. The rationale for the ensemble was that the five individual network architectures made slightly different errors, and therefore the mean output values served to smooth out these unique anomalies.

This study enabled the creation of an extremely high-quality data set. The method of composite medical follow-up using CT and surgical notes as superior ground truth tests, combined with enhanced review of indeterminate cases, is arguably a more robust labelling method than has been reported elsewhere. Requests for use of the data set and trained models will be considered (by correspondence to the lead author) particularly for the purposes of further academic research.

Interestingly, this study demonstrated that amplifying the training data via image augmentation did not yield improved results. This contradicts the findings of work published elsewhere^32^ and suggests that image augmentation may only be effective in certain circumstances. This is likely related to the number of training images in the sample and how well these represent the true variation in the whole category population. If there are large differences between the training and test sets, there will be a tendency for overfitting which will be magnified by augmentation of the training data.

### Limitations

A major issue limiting the clinical relevance of these findings is the fact that abdominal radiographs are less sensitive than CT for diagnosis of small bowel obstruction.^33^ Furthermore, in some institutions CT has completely superseded the plain film as the first-line test. However, it is not clear whether this trend will continue, particularly if the demand for CT continues to outpace capacity in institutions with chronic radiologist vacancies. Plain radiographs are also likely to continue to play an important role in more resource limited settings, such as in developing countries.

It is also essential to point out that a single abdominal X-ray is never the sole basis for a diagnosis of small bowel obstruction. The findings are always considered within the wider clinical context with reference to multiple complex factors including clinical history, examination findings, lab tests and other imaging modalities. An AI system such as proposed here would therefore have to be limited in scope to the role of an alert system, merely flagging high risk cases for urgent clinical review. This system would never be used as the sole mechanism for making the diagnosis.

It is essential to point out that the accuracy metric presented here does not represent the accuracy of a combined AI and plain abdominal X-ray system in diagnosing small bowel obstruction clinically. It actually reflects the ability of the neural network to provide a result that agrees with the current gold-standard which is currently the consultant radiologist opinion. The inherent diagnostic limitations of plain abdominal radiographs are therefore unchanged. The enhanced labelling process which included review of a superior imaging modality and surgical outcomes could in theory be used to improve on this existing gold-standard but would ideally be evaluated in the context of a prospective clinical trial where outcome measures could be more accurately observed and comparison with radiologist performance more objectively assessed.

The exclusions from the data set mean that the network cannot be applied to all abdominal radiographs performed in clinical practice. However, relevant cases could be selected via computational analysis of the request form or via manual ‘opt-in’ by the requesting clinician. Further work is also necessary to adequately equip the network for cases of large bowel obstruction and indeterminate cases.

The neural networks in this study were trained on data with equal numbers of cases in each category (*i.e.* balanced data set of normal and abnormal cases). This method was selected because networks trained on unbalanced data sets typically demonstrate positive bias towards the category with the largest group. This is an ongoing area of research and solutions have been suggested to counteract this phenomenon, however in the experience of the authors, none of these solutions perform better than simply balancing the group sample size. The obvious limitation from using this strategy is that not all of the available data from normal abdominal X-rays could be used in training. Moreover, since balanced test sets were also used, the performance metrics are likely to be lower in clinical practice where the incidence of small bowel obstruction is lower. This would vary between institution and clinical setting and therefore more work is needed to evaluate the performance in specific clinical environments. Furthermore, the theoretical impact of an AI solution on patient outcome should also be validated via a prospective clinical trial before integration into the patient pathway.

In summary, this study has shown that AI in the form of deep learning can be used to identify small bowel obstruction on plain abdominal radiographs with high accuracy. This study suggests that an AI solution such as this could be used for almost instantaneous identification of high-risk cases at the time of image acquisition, flagging the case for expedited clinical review. Further prospective trials in the clinical setting may be justified to evaluate the impact on clinical outcomes.
