# Supplementary material for: An artificial intelligence deep learning model for identification of small bowel obstruction on plain abdominal radiographs
Source: Br J Radiol. 2021 Apr 27;94(1122):20201407. doi: 10.1259/bjr.20201407 (PMC8173678; doi:10.1259/bjr.20201407)
Supplement: Supplementary Material 1. [file bjr.20201407.suppl-01.docx]

| **Model** | **Optimiser** | **Learning rate** | **Batch size** | **Other** |
| --- | --- | --- | --- | --- |
| **VGG16** | Adadelta | 0.000002 | 20 | - |
| **Densenet121** | Adamax | 0.0001 | 20 | - |
| **NasNetLarge** | Adamax | 0.0001 | 16 | - |
| **InceptionV3** | SGD* | 0.00001 | 20 | Momentum = 0.9,  Nesterov = True |
| **Xception** | Adadelta | 0.1 | 20 | - |

**Supplementary File 1**

Hyperparameters details

^a^Stochastic Gradient Descent

**Keras ‘model.fit()’, callbacks:**

EarlyStopping(monitor='val_loss', patience=30, verbose=0, mode='min')

ModelCheckpoint('.mdl_wts.hdf5', save_best_only=True, monitor='val_loss', mode='min')

ReduceLROnPlateau(monitor='val_loss', factor=0.66, patience=14, verbose=1, epsilon=1e-4, mode='min', min_lr= 0.0000001^)

^^^This variable altered between networks depending on the initial learning rate.
