# Supplementary material for: An artificial intelligence deep learning model for identification of small bowel obstruction on plain abdominal radiographs
Source: Br J Radiol. 2021 Apr 27;94(1122):20201407. doi: 10.1259/bjr.20201407 (PMC8173678; doi:10.1259/bjr.20201407)
Supplement: Supplementary Material 2. [file bjr.20201407.suppl-02.pptm]

## Slide 1
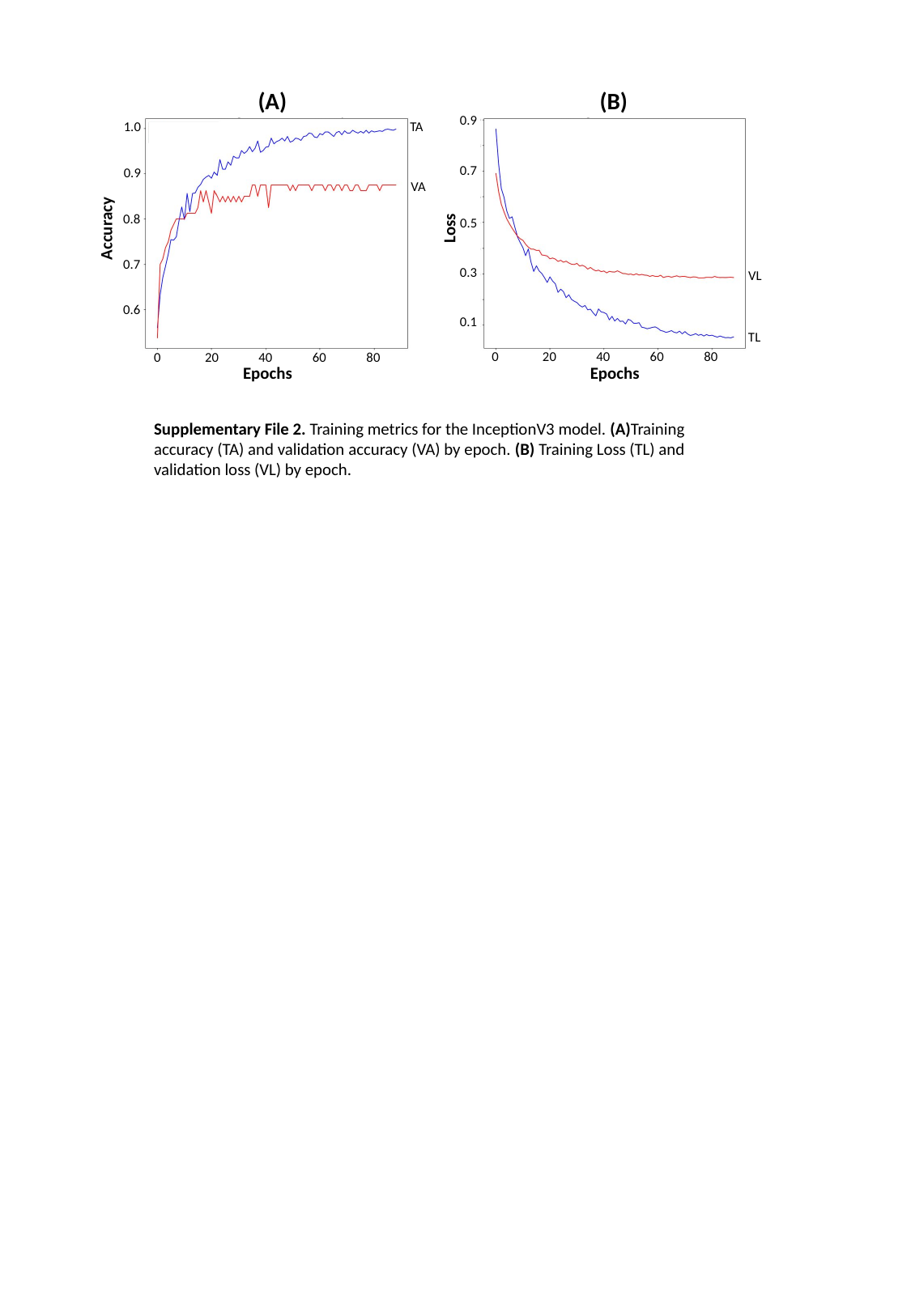

(A)
(B)
0.9
1.0
TA
0.7
0.9
VA
Accuracy
0.8
Loss
0.5
0.7
0.3
VL
0.6
0.1
TL
0
20
40
60
80
0
20
40
60
80
Epochs
Epochs
Supplementary File 2. Training metrics for the InceptionV3 model. (A)Training accuracy (TA) and validation accuracy (VA) by epoch. (B) Training Loss (TL) and validation loss (VL) by epoch.
